# Early Childhood Precursors and School age Correlates of Different Internalising Problem Trajectories Among Young Children

**DOI:** 10.1007/s10802-015-0116-6

**Published:** 2016-01-08

**Authors:** Alison Parkes, Helen Sweeting, Daniel Wight

**Affiliations:** MRC/CSO Social and Public Health Sciences Unit, University of Glasgow, Top floor, 200, Renfield Street, Glasgow, G2 3QB UK

**Keywords:** Internalising, Longitudinal, Trajectories, Child development

## Abstract

It is unclear why trajectories of internalising problems vary between groups of young children. This is the first attempt in the United Kingdom to identify and explain different trajectories of internalising problems from 46 to 94 months. Using both mother- and child-reported data from the large Growing Up in Scotland (GUS) birth cohort (*N* = 2901; male *N* = 1497, female *N* = 1404), we applied growth mixture modelling and multivariable multinomial regression models. Three trajectories were identified: low-stable, high-decreasing and medium-increasing. There were no gender differences in trajectory shape, membership, or importance of covariates. Children from both elevated trajectories shared several early risk factors (low income, poor maternal mental health, poor partner relationship, pre-school behaviour problems) and school-age covariates (low mother-child warmth and initial school maladjustment) and reported fewer supportive friendships at 94 months. However, there were also differences in covariates between the two elevated trajectories. Minority ethnic status and pre-school conduct problems were more strongly associated with the high-decreasing trajectory; and covariates measured after school entry (behaviour problems, mother-child conflict and school maladjustment) with the medium-increasing trajectory. This suggests a greater burden of early risk for the high-decreasing trajectory, and that children with moderate early problem levels were more vulnerable to influences after school transition. Our findings largely support the sparse existing international evidence and are strengthened by the use of child-reported data. They highlight the need to identify protective factors for children with moderate, as well as high, levels of internalising problems at pre-school age, but suggest different approaches may be required.

**Electronic supplementary material:**

The online version of this article (doi:10.1007/s10802-015-0116-6) contains supplementary material, which is available to authorized users.

In recent years it has been recognised that there are groups of children with distinctly different trajectories of internalising problems from early childhood to adolescence. Studies over the primary school years differ in the total number and shape of trajectories found: nonetheless, some common features have emerged. All studies show a stable low internalising problem trajectory (most children), and a consistently high trajectory (O’Connor et al. 2011; Sterba et al. 2007; Toumbourou et al. 2011; Weeks et al. 2014). Of these studies, all except one (O’Connor et al. 2011) also identified a trajectory that starts to increase at around 4–6 years from an initially moderate level. Importantly, current research on trajectories of internalising problems, and on subsets of these (anxiety and depressive symptoms), shows that childhood trajectories based on parent-reported problems help to predict self-reported pre-adolescent and adolescent depressive symptoms. All elevated trajectories predict greater likelihood of pre-adolescent or adolescent depression compared to the risk posed by a stable low childhood trajectory, but a greater risk appears to come from problems that persist at high levels or increase, as compared to more moderate or decreasing problems (Dekker et al. 2007; Feng et al. 2008; Sterba et al. 2007; Toumbourou et al. 2011). Early detection of internalising problems and the efficacy of early interventions to reduce adolescent depression and anxiety are likely to improve with a better understanding of unique as well as shared determinants of different trajectories. However, knowledge of factors associated with the development of different childhood trajectories is currently limited to a handful of studies, with little clarity regarding factors distinguishing different elevated trajectories (Cote et al. 2009; Letcher et al. 2009; O’Connor et al. 2011; Sterba et al. 2007; Weeks et al. 2014).

The presence of different elevated trajectories of internalising problems in childhood might reflect differences in overall burden of early risk or different constellations of early risk factors. The very few existing studies suggest elevated childhood internalising trajectories share early life predictors relating to the family system and child characteristics, including maternal depression, family dysfunction and stress, negative parenting, attachment problems, shy or irritable child temperament and child behaviour problems (Cote et al. 2009; Letcher et al. 2009; O’Connor et al. 2011; Sterba et al. 2007; Weeks et al. 2014). Within this matrix of early risk factors, there is some evidence that higher levels of risk predict the most elevated trajectories, but findings are sparse and inconsistent. One study confined to early childhood (1–5 years) found children in the most elevated trajectory were more likely to have a difficult temperament at 5 months old, or a mother suffering from depression, compared to children in a more moderate trajectory (Cote et al. 2009). However, a second study covering a later period (2–11 years) found maternal depression during infancy failed to distinguish between elevated trajectories (Sterba et al. 2007). Another study found greater socio-economic disadvantage and higher levels of infant behaviour problems both helped to differentiate increasing from decreasing trajectories, although these authors did not consider the role of maternal depression (Letcher et al. 2009). A more rounded picture of the contribution of early child problems, family dysfunction, maternal depression and socio-economic disadvantage to different internalising trajectories is required, to ascertain differences in the patterning and level of early risk factors for different elevated trajectories.

Elevated trajectories may be more easily distinguished by their correlates later in childhood. Researchers have hypothesised that the internalising problems of children following a consistently high trajectory may be relatively entrenched and difficult to modify, reflecting a high burden of early life risk centred on dysfunctional family processes. In contrast, children following intermediate trajectories may be more affected by environmental stressors and/or protective influences occurring after the early years (Letcher et al. 2009; Sterba et al. 2007). These later influences, coming increasingly from outside the family, might serve to escalate, or ameliorate, problems among children who already have some degree of early vulnerability. Such factors are likely to be particularly critical in transition periods, such as entry to primary school (Rimm-Kaufman and Pianta 2000).

To date, the hypothesis of later environmental vulnerability for intermediate trajectories remains largely untested, with only limited evidence for the role of school experiences. One study of internalising problems from 6 to 10 years found no main effects of school environment on trajectory class membership (O’Connor et al. 2011). Another found worse behavioural problems, school adjustment and social skills among 5–7 year olds from both increasing *and* consistently elevated internalising trajectories (Letcher et al. 2009). This study did not explore whether covariates around the time of school transition were more salient for intermediate groups than for the consistently elevated group. More research is needed to ascertain the involvement of school and other later influences in the shaping of intermediate increasing or decreasing trajectories, over the potentially vulnerable period of school entry.

A further limitation to existing work on diversity in the development of internalising problem trajectories is uncertainty over gender-specific pathways. Although the research literature suggests a female preponderance of adolescent affective disorder (Zahn-Waxler et al. 2008), during middle childhood (6–12 years) gender differences in trajectory shape and/or membership of different trajectory classes are inconsistent across studies (Sterba et al. 2007; Toumbourou et al. 2011; Weeks et al. 2014). Where gender differences in trajectory shape or membership exist, it is especially important to look for gender differences in trajectory covariates. Nevertheless, three studies examining this issue (Letcher et al. 2009; Sterba et al. 2007; Weeks et al. 2014) indicated relatively few gender differences, together with contradictory findings where differences appeared. One found that family dysfunction, including hostile or ineffective parenting, and behavioural problems were more predictive of elevated trajectories for females than for males (Weeks et al. 2014), while another found no gender differences in the influence of parenting but suggested behavioural problems were more important for boys (Letcher et al. 2009).

The current study investigates trajectories of mother-reported children’s internalising symptoms over the transition to primary school (from 46 to 94 months – approximately 4 to 8 years), using a large birth cohort study. These mother-reported trajectories are validated using child-reported measures at 94 months. Based on common findings from previous research (Sterba et al. 2007; Toumbourou et al. 2011; Weeks et al. 2014), we expect to find three trajectories: low stable, consistently high, and a trajectory increasing from an initially moderate level. From research using a time period most similar to our own (Sterba et al. 2007), we do not expect to find more than three. We examine trajectory covariates as possible influences on trajectory development, bearing in mind the important caveat that causality cannot be assumed. Overall trajectory shape is likely to reflect multiple influences over the entire developmental period studied. Although early life covariates appear to have clearest predictive value, they may not suffice once the child starts to be influenced by factors beyond the immediate family. In line with some previous research (Letcher et al. 2009; O’Connor et al. 2011) we also examine later covariates around and after school entry. With later covariates it is particularly important to stress uncertainty over the direction of association and causal mechanisms that may be involved. Nonetheless, and especially when we observe a trajectory with a pronounced change in slope after school entry, later covariates may provide valuable suggestions for processes contributing to overall trajectory shape.

Although existing research points to the existence of common factors underlying the development of elevated trajectories, we put forward two hypotheses to differentiate these.

1. The highest trajectory during the pre-school years will reflect a greater burden of early risk.
2. The role of later covariates will vary according to pre-school problem levels. In this regard, we expect that children with high pre-school problems will be relatively immune from further environmental change: in comparison, we expect to see greater change in the lives of children with moderate pre-school problems after they begin school.

With regard to covariate selection, we are guided both by the findings of existing studies of trajectory heterogeneity (Cote et al. 2009; Letcher et al. 2009; O’Connor et al. 2011; Sterba et al. 2007; Weeks et al. 2014), as well as by other longitudinal research on overall levels of childhood internalising problems. This has pointed to family factors including socioeconomic disadvantage (Flouri et al. 2014), father absence (Bayer et al. 2012) and minority ethnic status (Atzaba-Poria and Pike 2005); maternal characteristics including depression (Goodman et al. 2011); poor partner relationship quality (Spence et al. 2002); attachment problems and poor parenting (Madigan et al. 2013; Shaw et al. 1997); and child characteristics including delayed language skills (Bornstein et al. 2013), poor adjustment to the academic and social environment at school (Ackerman et al. 2007; van Lier et al. 2012); conduct and attentional problems (Meinzer et al. 2014; Wolff and Ollendick 2006); and low prosocial behaviour (Nantel-Vivier et al. 2014).

We also investigate gender differences in trajectory shape, class membership and the importance of covariates, although we expect to find few, and relatively small, differences over the age group studied.

## Method

Data were from the first birth cohort of the Growing Up in Scotland study, a nationally representative cohort of families with children born between June 2004 and May 2005. A brief description of the study is provided here: details of the sampling frame and methods are available elsewhere (Bradshaw et al. 2007). Baseline data were gathered from 5217 families during 2005–6, when children were 10 months old, and these families were followed up annually for 5 years (to 70 months), and then after 2 years (94 months, *N* = 3456, male *N* = 1747 female *N* = 1709). This study used data from computer-assisted personal interviews conducted with the main carer at 10, 22, 46, 58, 70 and 94 months, and data collected from the cohort child using a computer-assisted self-completion questionnaire at 94 months. It was restricted to cases where the natural mother and child were both interviewed at 94 months (exclusions, *n* = 77) and the natural mother was interviewed at all previous time points (further exclusions, *n* = 378) resulting in an analysis sample of 2901 cases (male *N* = 1497, female *N* = 1404). Each sweep of data collection was subject to medical ethical review by the Scotland ‘A’ MREC committee, with mothers or carers giving informed consent.

### Measures

Unless otherwise stated, information used was gathered from mothers. Computer-assisted self-completion modules were used for sensitive information regarding child behavioural and emotional problems, maternal health and partner relationships.

#### Main outcome measure: internalising problems

These were assessed using the Strengths and Difficulties Questionnaire (SDQ) (Goodman 1997, 2001). Items ask for agreement with statements concerning the child, with responses on a 3-point scale: (*0*) not true, (*1*) somewhat true, (*2*) certainly true. The combined five-item peer relationships and five-item emotional problems subscales produce an internalising symptoms scale ranging from 0 to 20, with good convergent and discriminant validity, both across informants and with respect to clinical disorder (Goodman et al. 2010). Measurements were made at four time points (46, 58, 70 and 94 months; Cronbach alphas respectively 0.61, 0.65, 0.70 and 0.76) .^1^

#### Child-reported well-being measures

Four aspects of children’s lives relating to subjective well-being were modelled as latent constructs, using information reported by the child at 94 months (loadings all ≥0.4, and factor determinacy scores all >0.8 indicating high internal consistency (Grice 2001). *Life satisfaction* used five items from Huebner’s Student Life Satisfaction Scale (Huebner 1991; Huebner and Alderman 1993) concerning whether the child felt he/she had a good life, had what he/she wants in life, his/her life is just right, wished life was different, and felt life was going well. *Positive parenting* used five items from the Alabama Parenting Questionnaire, relating to parental use of positive reinforcement and interaction with the child (Elgar et al. 2007; Scott et al. 2011), for example “My parents tell me if I behave well”, “My parents play games or do other fun things with me”. *Liking school* and *supportive friendships* respectively used items from the school and friends domains of the Multidimensional Life Satisfaction Scale (Huebner 1994). *Liking school* used three items (“I enjoy learning at school”, “I hate school”, “I look forward to going to school”), while *supportive friendships* used two items (“My friends are mean to me”, “My friends are nice to me”).

Measures of potential covariates of internalising trajectories are outlined below with scale reliability for continuous scores (general information on reliability and validity is available in the cited references).

#### Child characteristics

*Developmental delay* was based on the *Communication and Symbolic Behavior Scales Developmental Profile* (CSBS DP) (Wetherby et al. 2002), identifying those with a total score below the recommended cut-off. *Conduct problems*, *attentional problems* and *prosocial behaviour* were reported at 46, 70 and 94 months using the five-item conduct problems, hyperactivity/inattention and prosocial subscales of the Strengths and Difficulties Questionnaire (Goodman 1997, 2001). Cronbach alpha values increased over the time period studied, ranging from 0.47 to 0.59 for conduct problems, 0.72 to 0.80 for hyperactivity/inattention and 0.62 to 0.66 for prosocial behaviour.

#### Socio-demographic characteristics, health and support

Maternal *ethnic group* was subdivided into white and minority (further subdivision was not possible due to the low prevalence of minority groups, 3 %). *Family income* (equivalised for household size and composition and divided into quintiles) and *absent father* (natural father not resident in the household) were based on baseline information (child aged 10 months).

*Maternal mental health* was assessed at baseline using the Short Form (SF-12) Health Survey scale (Jenkinson and Layte 1997; Ware et al. 1996). Poor mental health was defined as a score 1 standard deviation or more below the mean score. *Partner relationship* was based on four items asked at 22 months from the Golombok Rust Inventory of Marital State (GRIMS) (Rust et al. 1990) (Cronbach alpha = 0.76), covering partner listening and sensitivity, and respondent feeling lonely or on the brink of separation. When there was no resident partner, we used a single item measured at 22 months on the quality of relationship with the non-resident father.

#### Parenting and school

*Mother*-*infant bonding* at baseline was measured using five items (alpha 0.63) from the Condon Maternal attachment scale (Condon and Corkindale 1998). Items related to how the mother felt when caring for the child, and comprised annoyance/irritation, incompetence/lack of confidence, resentment, feeling down/low, and not being a good parent. *Smacking* was assessed using a single item at 22 months, asking whether the mother had ever smacked the child (yes/no response). School age parenting measures comprised *mother*-*child warmth and conflict* using the Pianta scales (Pianta 1992), assessed at 58 and 94 months (alphas at each age for warmth respectively 0.65, 0.68 and conflict 0.82, 0.85). Warmth was measured using eight items, for example: “(Child’s name) values his/her relationship with me”. Conflict was measured using seven items, for example: “(Child’s name) and I always seem to be struggling with each other”. *School maladjustment* used information gathered at the interview following school entry (either 58 or 70 months) and again at 94 months, and was based on items devised specifically for this study (alpha at each age 0.66, 0.51) concerning how often the child complained about school, looked forward to school, was reluctant to go, or said good things about school.

### Analysis

Simultaneous investigation of trajectories and covariates in mixture modelling presents considerable disadvantages associated with cumbersome model-building and selection (Asparouhov and Muthén 2014; Vermunt 2010). Consequently, our analysis was conducted in two separate stages: growth mixture modelling (GMM) using Mplus version 7.3 (Muthén and Muthén 1998–2012) to identify the number and shape of different trajectories of internalising symptoms, followed by exploration of child-reported outcomes and trajectory covariates using Mplus and Stata SE/13.1 (StataCorp 2013) respectively.^2^ Both stages took account of the complex survey design and used weights to counteract the effects of differential attrition.

Following recommended practice (Ram and Grimm 2009), a single-class latent growth curve model of internalising problems was first created (with significant positive intercept, negative linear and positive quadratic terms), before exploring trajectory composition using a staged procedure. An initial set of models was directed at confirming our hypothesis of three trajectories. This allowed mean growth parameters to vary between classes while variances and covariances were constrained to be equal across classes. Various model fit statistics were used to help identify the optimum number of classes, together with considerations of the smallest class size and posterior probabilities of class membership (Jung and Wickrama 2008). Smaller Akaike Information Criteria (AIC) and Bayesian Information Criteria (BIC) values are preferable, while Entropy values should be close to 1. The Lo, Mendell and Rubin Likelihood Ratio Test (LMR) test indicates whether a model has a better fit than the model with one fewer class.^3^ Further models explored the consequence of relaxing the equal class variance constraint. Gender differences in trajectory growth parameters were compared within the same model, using a multiple group framework. For GMM, missing outcome data were handled using Full Information Maximum Likelihood.

After saving trajectory class membership, we explored associations between trajectory class and the four child-reported well-being measures (modelled jointly), in order to assess whether child-reported information validated mother-reported internalising problems. We then explored associations between trajectory class and covariates. Missing information for covariates was generally at low levels (between 0 and 2.5 % for most items). Nonetheless, the cumulative effects of missing information meant that a complete case analysis (*N* = 2292) contained 29 % fewer cases than the full sample. To reduce bias and increase statistical power, missing data were imputed using Stata’s chained equations facility, creating twenty imputed data sets with results combined across these. Multinomial regression was used to model associations between covariates and trajectory class membership. Results for this type of regression are reported as relative risk ratios (RRR), where the odds associated with a risk factor for membership in any one of a number of trajectory groups is calculated relative to the odds for whichever group is selected as the reference group. Contrasts are reported as significant where probability *p* < 0.05. Two sets of comparisons were made: the first contrasted elevated trajectories with the low stable class, while the second compared elevated trajectories directly with one another. A series of models was used to explore early and late correlates of trajectory class membership. The first examined early life factors measured at infancy and toddlerhood. Then, based on data availability at later time points, we divided analysis of later covariates into two sets of further models. One set explored child behaviour over four time points (from 46 to 94 months), and the other explored mother-child relationship and school adjustment over two time points (school entry at 58/70 months, and 94 months). Both sets adjusted for early life factors, and the second set also adjusted for child behaviour at pre-school age (46 months). Gender differences in associations between covariates and trajectory class were tested by modelling interactions between gender and statistically significant (*p* < 0.05) trajectory covariates.

## Results

Sample information is provided in Table 1.

**Table 1:** Sample Information, *N* = 2901

Concept	Measure	Time point	Response category or range	% or mean (standard error)
Child characteristics	Child gender	10 months	male	51.6
			female	48.4
	Developmental delay	22 months	no	92.6
			yes	7.4
	Internalising problems	46 months	0 to 17	2.36 (0.06)
		58 months	0 to 14	2.32 (0.06)
		70 months	0 to 18	2.29 (0.07)
		94 months	0 to 18	2.77 (0.08)
	Conduct problems	46 months	0 to 9	1.98 (0.03)
		58 months	0 to 10	1.75 (0.03)
		70 months	0 to 10	1.60 (0.04)
		94 months	0 to 10	1.54 (0.04)
	Hyperactivity/inattention	46 months	0 to 10	3.66 (0.05)
		58 months	0 to 10	3.72 (0.05)
		70 months	0 to 10	3.57 (0.05)
		94 months	0 to 10	3.53 (0.05)
	Prosocial behaviour	46 months	0 to 10	7.85 (0.04)
		58 months	0 to 10	8.25 (0.04)
		70 months	0 to 10	8.43 (0.03)
		94 months	1 to 10	8.52 (0.03)
	School maladjustment	58/70 months	−0.59 to 3.65	0.02 (0.02)
		94 months	−0.45 to 3.75	0.05 (0.03)
Maternal characteristics	Mother’s ethnic group	10 months	white	97
			minority	3
	Mother’s age at birth of child	10 months	Under 20	7.5
			20 to 29	41.6
			30 to 39	47.8
			40 or older	3.1
	Low maternal mental health	10 months	no	84.8
			yes	15.2
	Stronger partner relationship	22 months	−3.30 to 1.25	0.00 (0.02)
Family characteristics	Father in household	10 months	yes	79.3
			no	20.7
	Number of children	10 months	one	47.7
			two or three	48.2
			four or more	4.2
	Family income	10 months	q1 (lowest)	21.9
			q2	20.2
			q3	18.5
			q4	21.1
			q5 (highest)	18.3
Parenting	Mother-infant bonding	10 months	−4.18 to 1.78	−0.01 (0.01)
	Smacking	22 months	no	83
			yes	17
	Mother-child warmth	58 months	−5.87 to 0.36	−0.04 (0.02)
		94 months	−5.93 to 0.46	−0.04 (0.01)
	Mother-child conflict	58 months	−0.94 to 2.58	0.04 (0.02)
		94 months	−0.90 to 2.71	0.04 (0.01)

### Number of Trajectories of Internalising Problems

**Fig. 1:**
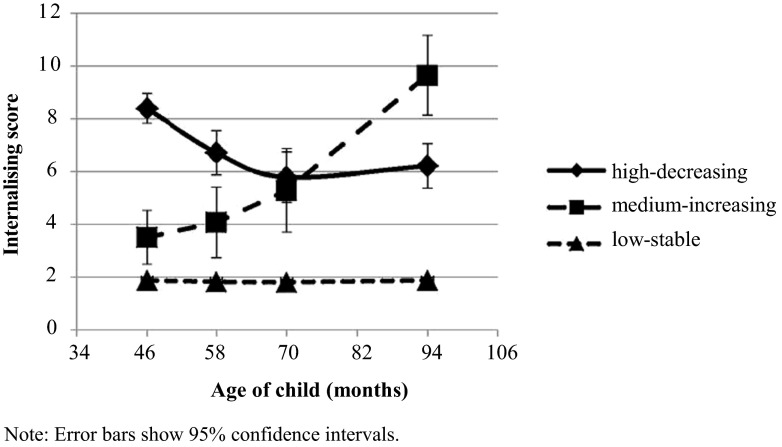
Trajectories of child internalising symptoms

Model fit indices from GMM of internalising problem trajectories are shown in Table 2 .^4^ AIC and BIC model fit statistics continued to fall, though less steeply, as the number of classes was increased from 1 to 4. With a four-class model, the lowest class size (3.5 %) and posterior probability (0.85) were adequate; however, this model was not significantly different from the three-class model using the LMR test and also had lower Entropy. As the more parsimonious three-class model also fitted with our expectations from prior research, we selected this for study here. Figure 1 shows the three trajectories: the majority of children (86 %) were in the low-stable trajectory, 6 % were in the high-decreasing trajectory and 8 % were in the medium-increasing trajectory. The high-decreasing trajectory and medium-increasing trajectory are here termed “elevated trajectories”, since mean scores were above the British mean of 3.3 for parent-reported internalising problems among children aged 5–10 years.^5^ Table 3 shows growth factor parameter estimates for the three-class model,^6^ as well as posterior probabilities. High values for these probabilities (0.9 or greater) indicate high classification accuracy. A comparison of male and female growth parameters for each trajectory class indicated no significant difference between the model where these were constrained to be equal, and a model where they were allowed to vary (Wald test = 4.02, 3° of freedom, *p* = 0.26). There was also no significant model difference when any one set of growth parameters was constrained to be equal across gender, and the remaining two were allowed to vary.

**Table 2:** Model selection and fit indices

Classes	Log likelihood	Free parameters	AIC	BIC	BIC (Sample-Size Adjusted)	Entropy	LMR *p* value for (k-1) classes
1	−24,695.3	13	49,416.61	49,494.25	49,452.95	^a^	
2	−24,234.91	17	48,503.82	48,605.36	48,551.35	0.913	0.005
**3**	**−24,012.61**	**21**	**48,067.23**	**48,192.66**	**48,125.94**	**0.924**	**0.049**
4	−23,845.44	25	47,740.89	47,890.21	47,810.78	0.892	0.236

Model with the best fit is shown in bold

*AIC* Akaike information criteria, *BIC* Bayesian information criteria, *LMR p* value, Lo, Mendell, and Rubin likelihood ratio test *p* value

^a^Not estimable for a 1-class model

**Table 3:** Growth factor parameter estimates and posterior probabilities for the best-fitting model

Trajectory (sample %)	Intercept		Linear slope		Quadratic slope		Posterior probability
	mean (SE)	*p*	mean (SE)	*p*	mean (SE)	*p*	
Low-stable (86 %)	1.89 (0.07)	**	−0.07 (0.06)		0.02 (0.01)		0.98
High-decreasing (6 %)	8.46 (0.55)	**	−2.13 (0.43)	***	0.40 (0.10)	***	0.90
Medium-increasing (8 %)	3.51 (0.30)	**	0.26 (0.34)		0.32 (0.08)	**	0.91

*SE* standard error

** *p* < 0.01, ***p < 0.001

### Associations Between Trajectory Classes and Child-Reported Outcomes

We examined how internalising trajectories based on mother-reported information related to child-reported measures at the trajectory end points (94 months). Compared with the low-stable trajectory, both elevated trajectories predicted less supportive friendships (Table 4). The high-decreasing trajectory predicted less positive parenting, while the medium-increasing trajectory predicted lower life satisfaction. Liking school did not differ significantly between trajectories. Differences between the two elevated trajectories (tested by re-setting the reference class, not shown) were not significant for positive parenting or supportive friendships, but lower life satisfaction for the medium-increasing trajectory bordered significance (*p* = 0.07). Inclusion of gender and gender interaction terms showed no gender differences in the associations found.

**Table 4:** Associations between internalising problem trajectory class and child-reported outcomes

Trajectory class (reference = low-stable)	Child-reported outcomes at 94 months							
	Life satisfaction		Positive parenting		Like school		Supportive friends	
	β	*p*	β	*p*	β	*p*	β	*p*
High-decreasing	−0.02		−0.07	*	0.02		−0.07	*
Medium-increasing	−0.12	***	−0.02		−0.05		−0.11	**

Outcomes were modelled jointly

* *p* < 0.05, ** *p* < 0.01, *** *p* < 0.001

### Trajectory Covariates

Unadjusted associations with trajectory class for child, maternal and family characteristics are provided in a supplementary file. These indicate significant differences between elevated trajectories and the low-stable trajectory with regard to almost all early life factors, but relatively few differences when directly comparing the two elevated trajectories. Pre-school (46 months) conduct problems were higher and prosocial behaviour lower in the high-decreasing compared to the medium-increasing trajectory. Examination of repeated measures available for covariates from pre-school age (46 months) suggests that adverse trends to 94 months were confined to the medium-increasing trajectory. For these children, attentional problems increased steadily, although conduct problems had a later onset. Mother-child relations and school maladjustment also worsened. In contrast, these covariates either improved or remained stable for the high-decreasing trajectory class.

Results of multivariable multinomial regression models are shown in Table 5. The first model (Set 1) considered early life factors. Here, membership of both elevated trajectories was predicted by lower family income, low maternal mental health and a poorer partner relationship. There were also some differences in factors predicting each elevated trajectory, when these were compared to the low trajectory. However, when the two elevated trajectories were compared directly only one contrast was statistically significant, namely maternal minority ethnic group status (less strongly associated with the medium-increasing trajectory). Father absence (more strongly associated with the medium-increasing trajectory) bordered significance (*p* = 0.05).

**Table 5:** Child, maternal and family characteristics associated with internalizing problem trajectory class: results of multivariable multinomial regression models

Correlate set modelled	Trajectory correlate	Trajectory class contrast					
		High-decrease v. Low-stable		Medium-increasing v. Low-stable		Medium-increasing v High-decreasing	
Set 1: Early life factors		RRR	*p*	RRR	*p*	RRR	*p*
10–22 months	Child female gender	0.62	*	0.77		1.25	
	Child developmental concern (yes)	2.29	**	1.38		0.60	
	Maternal minority ethnic group	3.17	***	0.51		0.16	*
	Family income (lower)	1.44	***	1.29	**	0.90	
	Father absence	1.16		2.20	***	1.89	†
	Low maternal mental health	2.19	**	1.86	**	0.85	
	Partner relationship quality (greater)	0.64	**	0.66	**	1.04	
	Mother-infant bonding (greater)	0.98		0.75	*	0.76	
	Smacking (yes)	0.99		1.50	†	1.52	
Set 2: School-age correlates							
A) Child behaviour							
46 months	Conduct problems	1.50	***	1.18	*	0.79	*
	Attentional problems	1.11	†	1.14	**	1.03	
	Prosocial behaviour	0.92		0.95		1.04	
58 months	Conduct problems	1.14		0.94		0.82	†
	Attentional problems	0.96		1.14	**	1.18	*
	Prosocial behaviour	0.90	†	0.91		1.01	
70 months	Conduct problems	0.91		1.17		1.28	*
	Attentional problems	1.10		1.23	**	1.11	
	Prosocial behaviour	0.98		1.02		1.04	
94 months	Conduct problems	1.02		1.38	***	1.35	**
	Attentional problems	1.03		1.27	***	1.23	*
	Prosocial behaviour	0.97		0.92		0.95	
B) Mother-child relations and school adjustment							
58/70 months^a^	Mother-child warmth	0.60	***	0.78	†	1.31	†
	Mother-child conflict	1.14		1.55	**	1.36	
	School maladjustment	1.28	*	1.31	*	1.02	
94 months	Mother-child warmth	0.82		0.82	†	0.99	
	Mother-child conflict	1.17		2.60	***	2.23	***
	School maladjustment	1.21		1.71	***	1.41	**

Analysis used imputed data set, *N* = 2901. Sets 2A and 2B models of later covariates show models built up at a succession of time points, adjusting for corresponding measures at all previous time points as well as adjusting for all early life covariates. Set 2B models also adjusted for child conduct problems, attentional problems and prosocial behaviour at 46 months

*RRR* relative risk ratio

^a^Warmth and conflict were measured at 58 months, and school maladjustment at 58 or 70 months, depending on school entry age

† *p* < 0.1, *p < 0.05, ** *p* < 0.01, ***p < 0.001

Later covariates were modelled in two groups (Sets 2A and B, Table 5). Set 2A considered child behaviour at 46, 58, 70 and 94 months by progressively adding measures at each time point to those included in the model for the preceding time point.^7^ All models in this set further adjusted for early life factors. For example, the 58 month model examined whether child behaviour at this time point predicted trajectory class membership, given the child’s behaviour at 46 months and early-life factors. At pre-school age (46 months), higher levels of conduct and attentional problems were found for both elevated trajectories (borderline for attentional problems in the high-decrease trajectory), but comparing the trajectories directly indicated lower conduct problems in the medium-increase trajectory. Adding child behaviour measures at 58, 70 and 94 months to successive models did not further distinguish the high-decreasing trajectory from the low group, but did continue to predict membership of the medium-increase group. Attentional problems at 58, 70 and 94 months were higher for the medium-increase than the low group, and conduct problems were higher at 94 months. A direct comparison of the two elevated trajectories showed higher attentional problems at 58 months, conduct problems at 70 months and both types of problem at 94 months in the medium-increasing trajectory.

A second set (2B) of later covariate models considered the mother-child relationship and the child’s adjustment to school, at 58/70 months and 94 months. Both models adjusted for early-life factors and child behaviour at 46 months. The 94 month model also included mother-child relationship and school maladjustment at 58/70 months. At school entry age (58/70 months), school maladjustment was higher while mother-child warmth was lower for both elevated trajectories (difference borderline, *p* = 0.05 for the medium-increasing trajectory), and conflict was higher for the medium-increasing trajectory only. No clear difference between elevated trajectories was apparent at this time point. Adding 94 month information in the second model in this set did not further distinguish membership of the high-decreasing, compared to the low-stable trajectory class. However, greater mother-child conflict and school maladjustment at 94 months distinguished the medium-increasing class from both the low-stable and high-decreasing classes.

Tests of gender differences by adding interaction terms to a multivariable model of early life factors and child behaviour at 46 months found an interaction with absent father was statistically significant when comparing the medium-increasing trajectory with the low-stable trajectory (0.42, *p* = 0.02). This indicated that absent father was less important for girls than boys in the medium-increasing trajectory: relative risk associated with absent father was 1.11 for girls, *p* = 0.75; and 2.65 for boys, *p* < 0.001. Inclusion of this interaction term in the model when comparing elevated trajectories directly indicated a significant difference in the likelihood of boys with an absent father being in the medium-increasing rather than the high-decreasing trajectory (relative risk 2.26, *p* < 0.05). The gender interaction term lost significance in models incorporating later covariates. Elsewhere, gender interaction terms were not significant.

## Discussion

This study, using a large birth cohort, identified three trajectories of internalising problems among young children from pre-school age until around 2 years after the transition to primary school: low-stable (the normative group), high-decreasing and medium-increasing. While studies extending to the teenage years have found a larger number of trajectories (Toumbourou et al. 2011; Weeks et al. 2014), our findings regarding number and general shape of the trajectories are most consistent with a US study of children from 2 to 11 years (Sterba et al. 2007). The medium-increasing trajectory found in our own study and that of Sterba et al. echoes the studies extending further into adolescence: however, in our study the two elevated trajectories intersected at an earlier age than found elsewhere. Sterba et al’s study differed from ours in finding gender differences in trajectory shape: these were small apart from a pronounced increase in internalising problems among girls in the late primary school period (7–11 years), which was not covered by our study. Gender was not a predictor of elevated trajectory class membership in our final model, although other research has found some (apparently contradictory) gender differences (Sterba et al. 2007; Toumbourou et al. 2011; Weeks et al. 2014).

The mother-reported trajectories found here were partially validated from child-reported data collected at the trajectory endpoint (approximately 8 years). Compared with the low-stable group, children in both elevated trajectories reported less supportive friendships, in agreement with mother-reported peer relationship problems contained within the SDQ scale of internalising problems used for this study. Children with elevated trajectories did not appear to differ from the low-stable group in their enjoyment of school, perhaps because academic aspects of school were less important for them than social ones. Only children in the high-decreasing trajectory reported less positive parenting, although mothers reported less warm relationships with children in both elevated groups. Children in the medium-increasing trajectory reported lower life satisfaction, which appears to tally with the higher level of internalising problems at the trajectory end point. Elsewhere, children’s life satisfaction has been shown to negatively covary with anxiety and depression (Proctor et al. 2009).

Our study adds to the limited existing evidence for shared early precursors of elevated internalising trajectories: these were low maternal mental health, low family income, poorer partner relationship during infancy and toddlerhood, together with pre-school age conduct problems. Other studies have also found maternal depression and family dysfunction (possibly related to partner discord) associated with all elevated trajectories, but findings in respect of low family income are less consistent (O’Connor et al. 2011; Sterba et al. 2007; Weeks et al. 2014). Pre-school age conduct problems were predictive of both elevated trajectories, in agreement with other studies finding effects of externalising problems in 2–4 year olds (Letcher et al. 2009; O’Connor et al. 2011; Weeks et al. 2014). Two of these (Letcher et al. 2009; Weeks et al. 2014) found independent effects for pre-school age conduct and attentional problems, also suggested by our study, although the effect of pre-school attentional problems was clearest for the medium-increasing group. Taken together, these findings are consonant with research suggesting links between early behaviour problems and overall levels of childhood internalising problems. This may reflect shared early life risk factors (Bayer et al. 2012); as well as pathways operating at a later stage, via poor academic achievement and peer rejection (Gooren et al. 2011; van Lier et al. 2012). Our (previously untested) finding that the level of pre-school conduct problems differentiated between the two elevated trajectories supports our first hypothesis in relation to the stronger effect of early risk for the most elevated trajectory.

In addition to shared early predictors for both elevated trajectories, there were some interesting differences in early life precursors distinguishing each of the elevated trajectories from the low-stable trajectory. In this regard, our findings do not conform readily to our second hypothesis that the highest trajectory during the pre-school years will reflect a greater burden of early risk, but suggest the possibility of different constellations of risk factors for different elevated trajectories. Only minority ethnic status emerged as significant in a direct comparison of the elevated trajectories, although statistical power may have limited the ability to detect differences between the two relatively small groups of children. Minority ethnic status has been found to predict more child mental health problems in some other UK research, where it has been linked to lower social support and negative parenting style (Atzaba-Poria and Pike 2005). The low prevalence of ethnic minority families in our sample meant we could not distinguish between different ethnic groups, and we lacked information on length of residence in Scotland. In addition, ethnic differences in the reliability and validity of the outcome measure may have contributed to our finding (Mieloo et al. 2014).

School-age covariates of both elevated trajectories were initial school maladjustment and lower mother-child warmth. Although our study cannot demonstrate causal effects, such findings add to current sparse evidence that a child’s attitudes to school and parent–child relationships help to characterise elevated internalising trajectories, in addition to behavioural problems at school entry (Letcher et al. 2009). Our finding for low mother-child warmth could reflect earlier attachment problems (Groh et al. 2012). Early vulnerability at home, in terms of poor maternal mental health and attachment problems, is also likely to play a role once a child starts to develop moderate levels of internalising symptoms. Research has pointed to a vicious cycle, whereby withdrawn or avoidant child behaviour fuels maternal depression and poorer parent–child relations, which in turn lead to further internalising problems (Ciciolla et al. 2014; Yan and Dix 2014).

After children started attending school, more differences between covariates associated with the two elevated trajectories emerged, supporting our second hypothesis (Letcher et al. 2009; Sterba et al. 2007). Greater mother-child conflict, school maladjustment and behaviour problems after this point clearly differentiated the medium-increasing from the high-decreasing trajectory, as well as from the low-stable trajectory. In contrast, later covariates did not help in further differentiation of the high-decreasing trajectory from the low-stable group, suggesting lower susceptibility to later environmental modifiers. Attentional problems already distinguished the medium-increase from the low trajectory at pre-school age, and grew further over the period of school transition, starting before a similar growth in conduct problems. An Australian study also found that hyperactivity and poor mother-child relations from 5 to 7 years differentiated increasing from decreasing internalising trajectories, albeit only for boys (Letcher et al. 2009). Other research highlights how attentional problems affect academic and social functioning (Diamantopoulou et al. 2007), leading to peer rejection and impaired social skills (Murray-Close et al. 2010), together with internalising symptoms (Carroll et al. 2005; Herman and Ostrander 2007). While it is important to re-iterate uncertainty over causation in our own study, particularly in the case of covariates measured at the trajectory endpoint, it seems possible that increases in attentional and conduct problems, school maladjustment and parent–child conflict may all contribute to escalating internalising problems for those in the medium-increasing trajectory.

One gender difference in the effect of trajectory covariates was found, indicating that the lack of a resident father was more important for boys than girls in the medium-increasing trajectory. Caution needs to be applied in interpreting this single finding in view of the number of possible differences explored, especially as it has not (to our knowledge) been reported elsewhere. Our largely null finding for gender differences is consistent with our finding of gender similarities in trajectory form and class membership over the period studied. It fits with existing research presenting few, and apparently contradictory, gender differences (Letcher et al. 2009; Sterba et al. 2007; Weeks et al. 2014); as well as with research indicating similar pathways to childhood internalising problems for boys and girls from early behaviour problems (Gooren et al. 2011; van Lier et al. 2012).

Our study has several strengths, notably use of a large, nationally representative birth cohort and well-validated measures of child behavioural and emotional problems, information on a wide range of potential trajectory covariates including rarely-studied school adjustment, and child-reported measures to supplement mother-reported information. To our knowledge, this is the first UK study of its type. Future data collection will allow exploration of how the childhood trajectories we have identified extend over later primary school years, and map on to adolescent depressive symptoms, as illustrated elsewhere (Sterba et al. 2007; Toumbourou et al. 2011). Some differences between this and other studies of trajectory heterogeneity may reflect the use of different outcome measures, the shorter developmental period investigated here and/or between-country differences, for example in the timing of entry to primary school and pre-school education. However, the inclusion of the peer problems subscale in our measure of internalising symptoms, as recommended for greater validity (Goodman et al. 2010), restricts comparability with some other studies based solely on measures of anxiety and depressive symptoms (Sterba et al. 2007; Toumbourou et al. 2011; Weeks et al. 2014). The use of a measure that includes peer problems is also a limitation when examining associations between trajectory class and child-reported supportive/unsupportive friendships. Additional study limitations stem from the number, timing and source of outcome and covariate measures. Having four repeated outcome measures restricted us to a model with linear and quadratic terms, although a piecewise model using more time points might better capture whether there are distinct structural processes involved before and after children start school. Later covariates do not have clear predictive value, since information measured after the start of trajectories may reflect, as well as influence, the development of internalising problems. Associations between trajectory class and covariates may be inflated by omitted variables and shared method variance due to reliance on mothers’ reports, resulting in uncertainty over the causal basis for associations found for early life as well as later covariates. Future work should incorporate information from different observers, including fathers and teachers; as well as more independent assessments and observations of child characteristics. It should include information on children’s social adjustment and academic progress in the early years of primary school, in order to distinguish more clearly between different possible routes to internalising problems, and incorporate more repeated measurement of covariates in order to study these as parallel processes.

Further work is needed to corroborate the suggestion made here of distinct, as well as shared, pathways to elevated childhood internalising trajectories. A person-centred, rather than variable-centred, approach might help clarify whether elevated trajectories have different clusters of early risk factors, as demonstrated in a recent study of childhood adversity subtypes in relation to adolescent depression (St Clair et al. 2014). Research is also required to explore potentially modifiable protective factors. Our study suggests early support may be important for children in both elevated trajectories. Interventions to promote sensitive, supportive parent–child relationships may give extra resilience to temperamentally anxious or depressed young children, or to children with behavioural problems (Buss and Kiel 2011; Kok et al. 2013; Muhtadie et al. 2013). However, our results suggest more attention might need to be paid to different elements of early risk, such as ethnic minority status or father absence.

Once children are at school, the teacher-child relationship is likely to be of key importance for both trajectories, particularly as children adapt to the novel environment in the first primary school years, since this may impact on both academic and social skills (Hughes and Kwok 2006; Pianta and Stuhlman 2004; Roorda et al. 2011). A close teacher-child relationship may be particularly advantageous for children with insecure attachment or early behavioural and emotional problems (O’Connor and McCartney 2007; O’Connor et al. 2011; Silver et al. 2005). Nonetheless, our study points to the possible danger of a narrow focus on children with the highest level of pre-entry internalising problems, who may be relatively easy to identify from more visible conduct problems. Children with more moderate levels of pre-entry internalising problems, who may present fewer difficulties in terms of classroom management, should not be overlooked at this stage. The study also suggests a need for identify problems before they start to escalate.

## Electronic supplementary material

Below is the link to the electronic supplementary material.

ESM 1 (DOC 74 kb)

## Footnotes

1 Reliability for the subscales in the analysis sample was lower than for the combined scale, with Cronbach alpha 0.49 -0.64 for peer relationship problems and 0.50 - 0.68 for emotional problems.

2 In principle, the entire analysis could have been conducted using the new integrated Mplus three-step approach to identify trajectories and examine correlates (Asparouhov and Muthén 2014), although in practice we encountered convergence problems with the covariate imputation model using this software. However, the high Entropy value (>0.9) for the trajectory model signalled that we did not require this integrated approach.

3 The complex sample did not permit use of the Bootstrap Likelihood Ratio Test (BLRT) (Nylund et al. 2007).

4 This Table is restricted to classes identified assuming equal class variance and covariance. Relaxing this assumption produced lower AIC and BIC values, but lower posterior probabilities and considerably reduced Entropy (respectively, values of 0.64, 0.60 and 0.69 in 2-class, 3-class and 4-class models). Such models were rejected, as inadequate Entropy values (< 0.8) suggested poor separation of classes and low confidence in class membership (Ram and Grimm 2009).

5 See http://www.sdqinfo.org/norms/UKSchoolNorm.html for more information.

6 A three-class model setting the non-significant growth parameters to zero for the low-stable and medium-increasing trajectories had similar model fit statistics (AIC 48068.43, BIC 48181.91, Entropy 0.92 and LMR *p* = 0.05). With little to choose between this and the Model in Table 1 without these constraints, we opted for the latter.

7 Multicollinearity was not found to be a concern for these or the second set of models, with mean variance inflation 1.70 and 1.35 respectively.
