# Supplementary material for: Early Childhood Precursors and School age Correlates of Different Internalising Problem Trajectories Among Young Children
Source: J Abnorm Child Psychol. 2016 Jan 8;44(7):1333–46. doi: 10.1007/s10802-015-0116-6 (PMC5007267; doi:10.1007/s10802-015-0116-6)
Supplement: Supplementary file 1 — (DOC 74 kb) [file 10802_2015_116_MOESM1_ESM.doc]

**Supplementary material**

**Univariate associations between covariates and trajectory class**

| **Group** | **Covariate** | **Trajectory class** | | | **Contrast** | | |
| --- | --- | --- | --- | --- | --- | --- | --- |
|  |  | Low-stable | High-decreasing | Medium-increasing | High-decreasing vs. Low-stable | Medium-increasing vs. Low-stable | Medium-increasing v. High-decreasing |
|  |  | % or mean (SE) | % or mean (SE) | % or mean (SE) | *p* | *p* | *p* |
| **Early-life factors** **(10-22 months)** | Child female gender | 50.3 | 62.4 | 57.3 | * |  |  |
|  | Child developmental concern | 6.2 | 19.6 | 12.4 | *** | * |  |
|  | Maternal minority ethnic group | 2.6 | 11 | 1.3 | *** |  | ** |
|  | Family income (10 months) - second highest quintile | 20.2 | 26.8 | 16.7 | ** |  |  |
|  | q3 | 18.9 | 17.1 | 15.6 | *** | * |  |
|  | q4 | 22.3 | 15 | 13.2 | *** | * | * |
|  | Lowest quintile | 20.2 | 4.5 | 8.3 | *** | *** |  |
|  | Father absence | 17.8 | 29.5 | 44.4 | ** | *** | * |
|  | Low maternal mental health | 12.6 | 29.6 | 31.7 | *** | *** |  |
|  | Partner relationship quality (greater) | -0.04 (0.02) | -0.32(0.09) | -0.24(0.08) | *** | *** |  |
|  | Mother-infant bonding (greater) | 0.03 (0.01) | -0.14 (0.06) | -0.30 (0.07) | ** | *** |  |
|  | Smacking (yes) | 16.1 | 18 | 25.6 |  | ** |  |
| **Child behaviour** |  |  |  |  |  |  |  |
| 46 months | Conduct problems | 1.84 (0.03) | 3.22 (0.20) | 2.64 (0.11) | *** | *** | * |
|  | Attentional problems | 3.45 (0.05) | 5.14 (0.21) | 4.78 (0.19) | *** | *** |  |
|  | Prosocial behaviour | 7.94 (0.04) | 6.92 (0.23) | 7.47 (0.17) | *** | ** | * |
| 58 months | Conduct problems | 1.63 (0.03) | 2.82 (0.17) | 2.29 (0.13) | *** | *** |  |
|  | Attentional problems | 3.52 (0.05) | 4.91 (0.25) | 5.05 (0.18) | *** | *** |  |
|  | Prosocial behaviour | 8.35 (0.04) | 7.37 (0.19) | 7.75 (0.16) | *** | *** |  |
| 70 months | Conduct problems | 1.46 (0.03) | 2.42 (0.19) | 2.48 (0.17) | *** | *** |  |
|  | Attentional problems | 3.30 (0.05) | 4.96 (0.23) | 5.33 (0.21) | *** | *** |  |
|  | Prosocial behaviour | 8.53 (0.03) | 7.56 (0.21) | 7.96 (0.13) | *** | *** |  |
| 94 months | Conduct problems | 1.35 (0.03) | 2.34 (0.17) | 2.99 (0.15) | *** | *** | * |
|  | Attentional problems | 3.22 (0.05) | 4.83 (0.24) | 5.80 (0.21) | *** | *** | ** |
|  | Prosocial behaviour | 8.65 (0.03) | 7.69 (0.22) | 7.68 (0.14) | *** | *** |  |
| **Parent-child relations and school** |  |  |  |  |  |  |  |
| 58/70 months | Mother-child warmth | 0.01 (0.01) | -0.54 (0.15) | -0.23 (0.07) | *** | *** | * |
|  | Mother-child conflict | -0.03 (0.02) | 0.41 (0.08) | 0.48 (0.07) | *** | *** |  |
|  | School maladjustment | -0.02 (0.02) | 0.33 (0.11) | 0.24 (0.08) | *** | *** |  |
| 94 months | Mother-child warmth | 0.01 (0.01) | -0.37 (0.09) | -0.34 (0.05) | *** | *** |  |
|  | Mother-child conflict | -0.05 (0.01) | 0.38 (0.09) | 0.78 (0.08) | *** | *** | ** |
|  | School maladjustment | -0.05 (0.02) | 0.38 (0.14) | 0.90 (0.13) | *** | *** | * |

a Statistical tests of differences were based on multinomial regressions of trajectory class membership. ^b^ Timing depended on age child started school. **p*<.05, ***p*<.01, ****p*<.001
